# Rush Medical College; Twenty-Third Anniversary

**Published:** 1866-02

**Authors:** 


### EDITORIAL.

### RUSH MEDICAL COLLEGE—TWENTY-THIRD ANNIVERSARY.

The session, which has just been brought to a close, has been one of unusual satisfaction to the Faculty, and, if we may judge by their expressions of gratitude and interest, to the students of the College also.

While the Faculty feel that they have performed their daily duties punctually, from the day on which the session opened, to the very day it closed, they must give the class credit for unusual promptness and regularity of attendance.

The classes for the past three years have been considerably larger than those of previous years. Of the class in attendance during the past session, numbering two hundred and ninety-three, there were ninety who passed the examination for the degree of doctor of medicine.

The *ad eunden* degree was conferred upon Albert H. Hoy, M. D., Wisconsin; W. Louis Rabe, M. D., Illinois; J. J. Brown, M. D., Wisconsin; W. Y. Leonard, M. D., Indiana. The Honorary degree of Doctor of Medicine, was conferred on Gerhard Christian Paoli, M. D., Chicago.

Laudatory notices of annual commencements are invariably to be expected as a matter of course, as can be attested by consulting the published accounts of such exercises in any Medical College for years past.

We have no doubt, every class which has attended the regular course of lectures in Rush Medical College, has been better in nearly every respect than any preceding class. And yet, the Alma Mater has no reason to turn in shame from even her older children, when she sees how many of them have acquitted themselves nobly in the conflict with "disease and ignorance." We believe the class which has just graduated is better, absolutely—possibly not relatively—than any that has ever yet left the College. One would naturally expect this, for even in our decade, with the increase of population, and the accretion of wealth in the Northwest, the colleges, schools and other means of securing an education have been wonderfully developed. With these the general culture and education in the young men, who present themselves as students of medicine, have become proportionately superior to those of former times. Their opportunities have been better; they enter upon their professional studies with the feeling, that, for this reason, more will be expected of them. What we have here said of this class, we hope may be said in truth of every future class. As the country, and as Chicago develops its wealth, increases its population, and multiplies the means of education, so will ever improve, we trust, the true culture and education of those who enter upon the study of medicine.

It must be a matter of interest to the friends of the College to know, that the Board of Surgeons and Physicians of Cook County Hospital (recently opened,) after a careful examination of all applicants for the position of Resident Surgeon of the Hospital, unanimously declared Dr. N. I. Quales, of the graduating class, as the successful competitor.

It is also worthy of notice, that the prize for superiority of anatomical preparations was awarded to Mr. R. F. Ludwig, a student of dentistry, in the office of Dr. W. W. Allport, who is better fitting himself for the practice of his profession by the careful study of medicine. Every effort should be made to encourage all students of dentistry to pursue a thorough course of medical studies in connection with those of their especial department of science.

The twenty-third anniversary of the College was celebrated on the evening of January 24th. As the large number of the class and of their friends could not be accommodated in the Lecture-room of the College, the exercises were held in one of the public halls of the city. The presence of ladies, and the discourse of fine music by the orchestra, added to the pleasures of the evening.

In absence of the President of the College, Prof. Brainard, the degrees were conferred by Prof. Freer.

The valedictory address of Prof. Allen needs but little comment. It will be read with pleasure and profit by practitioners of long experience, as well as by those who are entering upon the more active duties of their profession. It was received with oft-repeated applause by the audience.

